# Mayaro Virus Pathogenesis and Transmission Mechanisms

**DOI:** 10.3390/pathogens9090738

**Published:** 2020-09-08

**Authors:** Cheikh Tidiane Diagne, Michèle Bengue, Valérie Choumet, Rodolphe Hamel, Julien Pompon, Dorothée Missé

**Affiliations:** 1 MIVEGEC, IRD, Univ. Montpellier, CNRS, 34394 Montpellier, France; michele.bengue@ird.fr (M.B.); rodolphe.hamel@ird.fr (R.H.); julien.pompon@ird.fr (J.P.); 2 Unité Environnement Risques Infectieux Groupe Arbovirus, Institut Pasteur, 75724 Paris, France; valerie.choumet@pasteur.fr

**Keywords:** Mayaro, emerging arbovirus, alphavirus, Togaviridae, Aedes, vector competence

## Abstract

Mayaro virus (MAYV), isolated for the first time in Trinidad and Tobago, has captured the attention of public health authorities worldwide following recent outbreaks in the Americas. It has a propensity to be exported outside its original geographical range, because of the vast distribution of its vectors. Moreover, most of the world population is immunologically naïve with respect to infection with MAYV which makes this virus a true threat. The recent invasion of several countries by *Aedes* *albopictus* underscores the risk of potential urban transmission of MAYV in both tropical and temperate regions. In humans, the clinical manifestations of MAYV disease range from mild fever, rash, and joint pain to arthralgia. In the absence of a licensed vaccine and clinically proven therapeutics against Mayaro fever, prevention focuses mainly on household mosquito control. However, as demonstrated for other arboviruses, mosquito control is rather inefficient for outbreak management and alternative approaches to contain the spread of MAYV are therefore necessary. Despite its strong epidemic potential, little is currently known about MAYV. This review addresses various aspects of MAYV, including its epidemiology, vector biology, mode of transmission, and clinical complications, as well as the latest developments in MAYV diagnosis.

## 1. Introduction

Following the major Zika virus (ZIKV) outbreaks in 2016, special attention has been given to neglected arboviruses. Arboviral infections are likely to spread globally as a result of the expansion of mosquitoes which, in turn, is caused by increased trade and climate change [1,2,3]. Mayaro virus (MAYV) is a single-stranded RNA virus. It belongs to the Togaviridae family and was first detected in 1954 in Trinidad in the sera of forest workers [4,5,6]. Like other alphaviruses, MAYV can infect, replicate and disseminate in both vertebrate and invertebrate hosts [7,8,9]. In humans, the virus causes Mayaro fever, which is characterized by long-lasting arthralgia similar to that occurring in Dengue fever. Although mortality among infected people is low as yet, Mayaro fever may become a major public health problem, particularly in rural areas, with increasing prevalence in the Amazon region due to ecosystem changes [10]. Although several outbreaks of Mayaro fever have already been reported more than three decades ago in Northern Brazil [11], MAYV is now spreading rapidly to other regions in Latin America [12]. It is likely that the global burden of Mayaro fever is still largely underestimated because of the lack of adequate and accurate diagnostics. At the same time, the *Aedes* mosquito, which can spread MAYV and other viruses, is invading new habitats and regions at an alarming pace [13]. Perhaps the most significant threat of a potential MAYV epidemic comes from increased colonization of urbanized areas by *Aedes aegypti*. In order to better predict the capacity of the virus to spread and infect new areas, further studies are urgently needed. At present, it cannot be excluded that MAYV infections, like those of ZIKV and Chikungunya (CHIKV) viruses, will one day become a major health issue. At present, MAYV is still neglected and few studies have been carried out on its pathogenesis, the biology of its potential vectors or the dynamics of its transmission. We present here a current and comprehensive review of published scientific research and technical reports on MAYV, focusing on its vectors, in addition to the epidemiological and clinical aspects of Mayaro fever.

## 2. Distribution and Significance to Health

**Figure 1:**
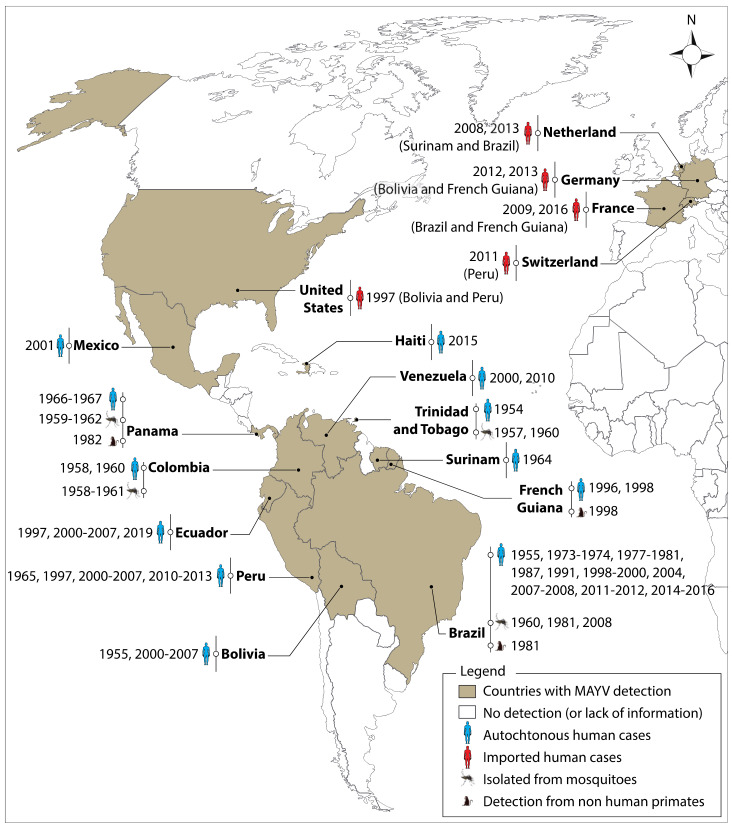
Global distribution of Mayaro virus (MAYV). The virus was detected in vectors, non-human primates and humans. The first imported cases were reported in the United States in 1997 and in Europe in 2008. The origin of imported cases is shown in parentheses. These reports do not exclude the possible presence of MAYV in other countries, notably Africa and Asia. MAYV is not necessarily present throughout the countries/territories shaded in this map. ArcGIS Desktop V 10.5 software was used to generate this map (ESRI 2019, Redlands, CA, USA: Environmental Systems Research Institute; https://desktop.arcgis.com/en/).

Mosquitoes are responsible for the transmission of a variety of deadly pathogens, including MAYV, that affect the lives and livelihoods of millions of people across the world each year. The global expansion of the two major urban vectors (*Ae*. *aegypti* and *Ae*. *albopictus*) and the increasing number of international travelers is certain to increase the risk of MAYV transmission [14]. There has been an underestimation of reported Mayaro fever cases reported since its first detection in 1954 which is not only due to its shared symptoms with Dengue and Chikungunya fever, but also to the occurrence of unreported asymptomatic cases [4,15,16]. Figure 1 illustrates the spatial distribution of the virus that was isolated from vectors, non-human primates (NHP) or humans. The presence of MAYV has been mainly reported in Latin America, with Brazil having the highest number of reported cases of Mayaro fever [4,6,10,17,18,19,20,21,22,23,24,25,26,27,28,29,30,31,32,33] and MAYV infections that were reported in Haiti indicate that the virus is also likely to circulate in the Caribbean [31,34]. A phylogenetic study carried out on whole-genome sequences of viruses isolated from Haitian patients suggests that the virus has been introduced into Haiti on three separate occasions [34]. Several imported cases of MAYV infections have also been reported from 1997 onwards in tourists returning from endemic areas of South and Central America, with several cases reported in Europe [14,35,36,37,38,39]. This scenario shows the importance of the potential role of air travel in the global spread of MAYV [14,35,36,37,38]. Due to a lack of information and reporting in Africa and Asia, it is as yet uncertain whether MAYV is present in these continents.

## 3. Clinical Manifestations and Pathogenesis of Mayaro Fever in Humans

**Figure 2:**
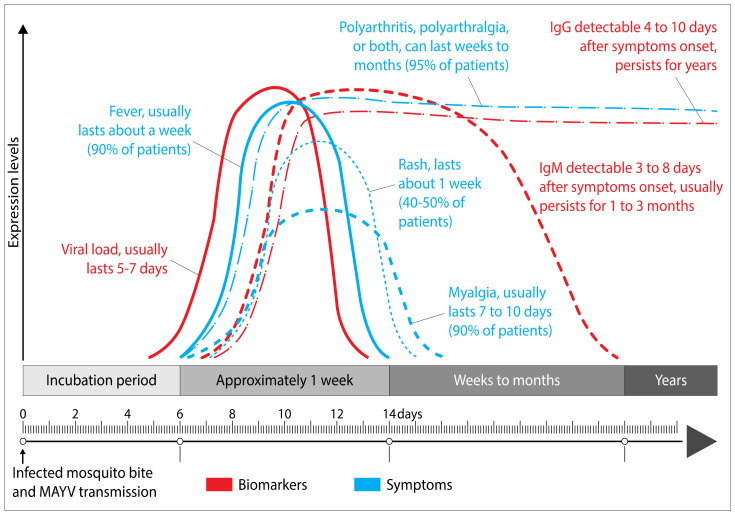
Pathogenesis of Mayaro fever. Time course of MAYV viremia and detection of immunoglobulin M/G (IgM/IgG) after the inoculation of MAYV into a susceptible host by an infected mosquito. Duration of clinical manifestations that may appear in an infected individual. The figure was designed using Adobe Creative Cloud apps (https://www.adobe.com/creativecloud.html).

**Figure 3:**
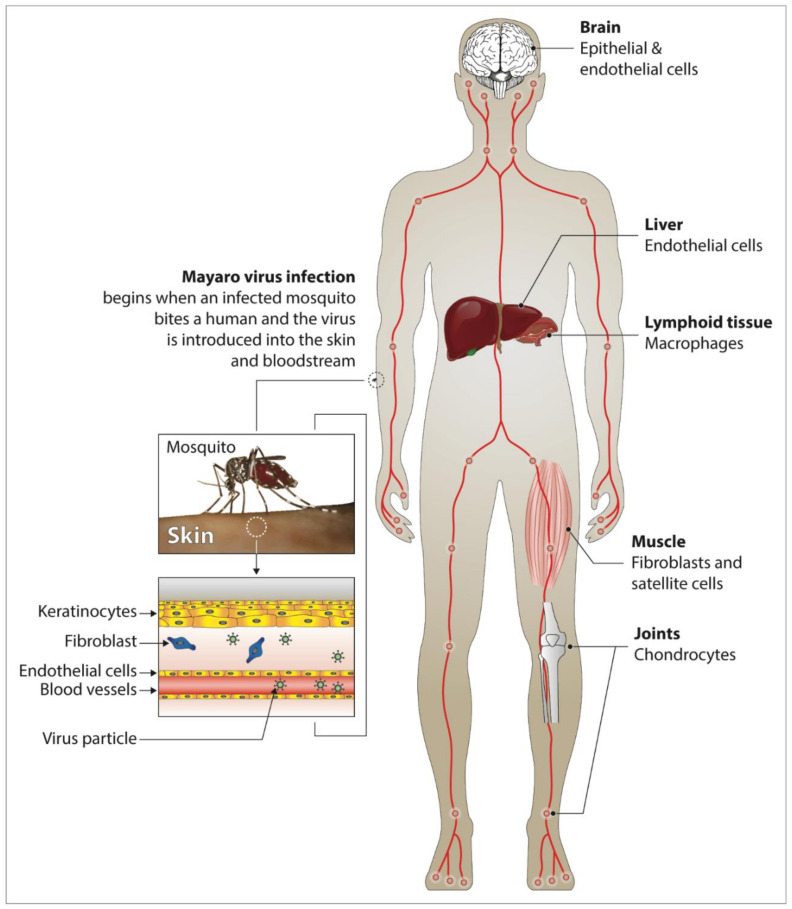
Diagram showing the probable dissemination of MAYV in humans, based on animal experiments and clinical data for similar alphaviruses. Transmission of MAYV occurs following its inoculation by an infected mosquito (*Haemagogus janthinomys*, *Aedes aegypti*, etc.). The virus then replicates in the skin (more precisely at the inoculation site of the virus by the competent vector) and propagates into the target tissues (muscle, the liver, joints, etc.) via the numerous blood vessels, followed by the recruitment of inflammatory cells in these tissues. Most of the target cells have not yet been identified for MAYV but the diagram shows an extrapolation based on other alphaviruses. The figure was designed using Adobe Creative Cloud apps (https://www.adobe.com/creativecloud.html).

Phylogenetically, MAYV is closely related to CHIKV and, like the latter, causes a debilitating flu-like illness in the infected host that is indistinguishable from Chikungunya fever. The main symptoms include chills, fever, gastrointestinal manifestations, eye pain, myalgia and arthralgia [18,29] (Figure 2). In particular, arthralgia can last for months to years, making the Mayaro fever even more debilitating than flavivirus infections, whose symptoms last only one to three weeks [4,11,15,40,41]. Acute symptoms generally last three to five days in most patients with Mayaro disease [15,25]. Arthralgia and myalgia account for 50–89% and 75% of infected patients, respectively [42]. Dizziness and itching are the other clinical manifestations of the disease [20,38]. Severe complications can occur due to MAYV infection, among which are myocarditis, hemorrhagic and neurological manifestations [17]. After a bite from an infected vector, MAYV spreads via the blood vessels into the body of the susceptible host (Figure 3). The virus replicates in white blood cells (e.g., monocytes, macrophages) and spreads to bones, muscles and joints via the main sites of replication, the spleen and the liver [15,43,44,45,46,47,48]. The severity of Mayaro fever is associated with the production of pro-inflammatory cytokines and mediators (MCP-1, IL-2, IL-9, IL-13, IL-7, VEGF, IL-17, and IP-10) both in humans and experimental mouse models [49,50,51]. Some of these cytokines have been shown to be associated with other pathologies involving bones and joints [47,52,53]. Mayaro fever can also induce oxidative stress (OS) in the liver of infected mice and in vitro infected HepG2 cells [54,55]. The induction of OS has also been observed in several other arbovirus infections, including Chikungunya [56,57,58]. The latter process is likely to play a role in the pathogenesis of MAYV, because of its important role in the initiation and control of the production of many soluble mediators such as reactive oxygen species (ROS), and is furthermore involved in apoptosis and inflammation [59]. Currently, little is known about the beneficial or detrimental effects of this process on the infected host. Many cell types have been shown to be implicated in the pathogenesis of MAYV. The role of macrophages, being targets for MAYV infection, in the development of arthritis has been demonstrated through the secretion of TNF-α and ROS [60]. In this respect, it is of interest to note that high serum levels of TNF-α have previously been reported in MAYV-infected patients and mice [50,61]. A recent study has also shown that in vitro infection of bone marrow-derived macrophages with MAYV results in the overexpression of key inflammasome proteins such as NLRP3, AIM2, ASC and caspase 1 [61]. Interestingly, the authors demonstrated that the induction of ROS was linked to the activation of the NLRP3 pathway. Because many studies have shown that ROS production can be associated with immune regulation [62], it would be of interest to investigate whether ROS secretion impacts the polarization of macrophages during MAYV infection. Results from studies using experimental mouse models and human cell lines have revealed that osteoblasts are susceptible to infection with alphaviruses, resulting in the secretion of MCP-1, IL-6, and IL-1β [43,63,64]. We have previously shown that primary human chondrocytes, osteoblasts and synoviocytes, which are the main cell types involved in arthralgia, are permissive to MAYV infection, indicating that these cells may be involved in the pathogenesis of the disease, notably as a result of the overexpression of arthritis-related genes [65]. Infection of the host by MAYV results in the sensitization of monocytes and the induction of their osteoclastogenic activity which leads, in turn, to bone erosion and cartilage damage. Taking into consideration the debilitating condition of MAYV-infected patients, which can last for months to years, further studies should be carried out to determine the mechanisms leading to severe arthritis as a result of MAYV infection. This also begs the question as to why some patients develop arthritis and others do not. It would also be interesting to develop studies leading to the identification of biomarkers or risk factors associated with severe forms of the disease. There is considerable variation in antibody production and persistence from one host to another. MAYV infection induces a transient production of immunoglobulin M (IgM) antibodies, indicating the occurrence of recent infection that generally lasts for at least three months after the onset of clinical symptoms [29,66,67] (Figure 2). In addition, the presence of virus-specific immunoglobulin G (IgG) serum antibodies that persists throughout the lifespan of an infected person is an indicator of prior infection with MAYV, in particular when present at increased levels [29,66,67]. Interestingly, a study by Santiago et al. shows that neutralizing antibodies alone are not sufficient to prevent the occurrence of chronic arthritis [50]. Earnest and collaborators generated a series of neutralizing broad-spectrum mouse antibodies by the use of recombinant MAYV E2 protein [68] and showed their therapeutic utility against MAYV, thereby underscoring the importance that should be given to IgG subclasses and their effector functions.

## 4. Diagnosis

The symptoms caused by MAYV infection are often difficult to be recognized because of the limited diagnostic laboratory facilities in many of the endemic regions. In addition to the variety of symptoms, which are not very specific, diagnosis of MAYV infection is rendered difficult by the existence of coinfections and the lack of rapid tests [25,69]. In MAYV surveillance, the discriminating diagnosis must also consider possible concomitant CHIKV, DENV, and ZIKV infections due to the co-circulation these viruses [20,31,33]. Neutralization and reverse-transcription polymerase chain reaction (PCR) tests are now available in almost all laboratories and can help in the confirmation of MAYV infection [31,38]. In a cross-sectional study in Ecuador, the prevalence of MAYV was estimated by evaluating the presence of IgM and IgG antibodies by ELISA [70]. It is of note that when samples are collected after the acute phase of clinical signs (which then no longer represents the viremia phase, lasting two to three days for MAYV), testing based on isolation is impractical. Nevertheless, this technique, which is generally performed in vivo in mice or in vitro by inoculation into cell cultures of arthropods (C6/36, Aag2, etc.) or vertebrates (Vero, BHK-21, etc.), remains the most accurate method for virus detection [20]. It is generally performed in vivo in mice or in vitro by inoculation into the cell cultures of arthropods (C6/36, Aag2, etc.) or vertebrates (Vero, BHK-21, etc.). In some circumstances, the isolation method is not the most appropriate due to several factors, including the costs involved and the need for additional tests, which increase the time required to deliver results. Complement fixation and neutralization tests are among the serological methods that can also be used in certain contexts for the diagnosis of MAYV. The detection of circulating IgM antibodies has the advantage of being specific and also constitutes the fastest technique. Indeed, the production of IgM usually occurs about five days after infection with MAYV [11,67]. For MAYV diagnosis, possible cross-reactions must be taken into account, in particular those between MAYV-, CHIKV- and O’nyong-nyong virus-specific antibodies [71,72,73,74]. In recent years, modern, more sensitive and faster methods using molecular biology techniques (i.e., PCR) have been widely used for the diagnosis of several pathogens of medical interest [75]. Accurate diagnosis of MAYV in human communities can also be rendered difficult due to the high prevalence of asymptomatic cases [25,31].

## 5. Genomic Structure and Viral Life Cycle

**Figure 4:**
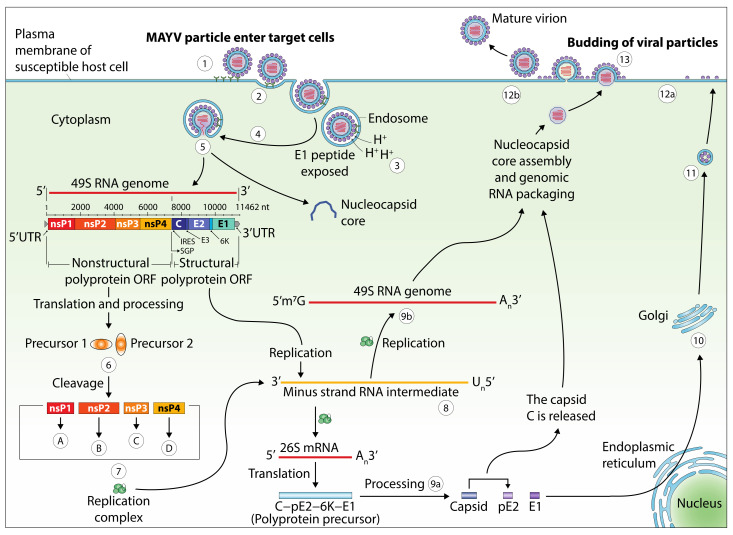
The replicative cycle and genomic structure of MAYV. E: envelope; nsP: nonstructural protein; UTR: untranslated region; nt: nucleotides. A: nsP1 is required in the formation of the viral RNA and the RNA capping processing; B: nsP2 presents several enzymes required in transcription; C: nsP3 is one element of the replication complex; D: nsP4 is known as the viral RNA polymerase. 1: endocytosis through a clathrin-coated pit or, alternatively, through a caveolin-coated pit; 2: start of virus internalization with receptors of the host cell; 3: the low pH obtained in the endosome leads to structural modifications in the envelope of the virus that reveal the E1. The latter mediates cell membrane-virus fusion; 4: endosomal cell membrane-virus fusion; 5: release of the capsid core and genome of the virus; 6: from the mRNA of the virus, a pair of nsP precursors are generated; 7: the replication complex is obtained from the gathering of nsP proteins. This complex thus allows the synthesis of a minus-strand RNA; 8: this RNA is used as the model to generate both genomic (49S) and subgenomic (26S) RNAs; 9a: processing of the polyprotein precursor by an autoproteolytic serine protease; 9b: genomic RNA involved in nucleocapsid core assembly and genomic RNA packaging; 10: processing and maturation of glycoproteins pE2 and E1; 11: in the Golgi, processed glycoproteins are associated. Then they are transported into the cell membrane; 12a: at the cell membrane, the pE2 is split into E2 and E3; 12b: association of the viral RNA with the capsid C and the recruitment of E1 allows viral assembly; 13: particles of MAYV associated with the core are released outside of the host cell through the membrane. The figure was designed using Adobe Creative Cloud apps (https://www.adobe.com/creativecloud.html).

The genome of MAYV, the causative agent of Mayaro fever, is a single-stranded RNA virus with positive polarity and a length of approximately 11.5 kilobases (kb). Its genomic structure forms two open reading frames, one coding for structural proteins and the other coding for non-structural proteins (Figure 4). The latter are expressed as a polyprotein that is cleaved both during and after translation into four non-structural proteins (nsP1, nsP2, nsP3, and nsP4), whereas the structural genes generate the six structural proteins (C, E1, E2, E3, 6K, and transframe) [71]. The viral particles are icosahedral, comprising a closed nucleocapsid in a compact envelope consisting of the host cell membrane sealed with E1-E2 complex [8,9,76]. The life cycle of MAYV starts with the fusion of the viral envelope with the plasma membrane of the target cells via specific receptors on their surface such as MXRA8, which has recently been revealed to be a receptor for several arthritogenic alphaviruses [7,77]. This fusion process, however, merits further study to determine the precise mechanisms involved. Next, the virus enters inside the cell by endocytosis and is subsequently found within the endosomal compartment. Once in the cytoplasm, the pH of the endosome decreases and the virus disassembles its basic structure and releases the genomic RNA which is translated into non-structural proteins that generate messenger RNA and various structural proteins. The latter are then transformed into a nucleocapsid and its glycoproteins, which, after combination with the plasma membrane, leads to the release of new viral particles outside the cell. The released particles then infect other cells and the cycle repeats itself [71].

## 6. Phylogeny

The complete nucleotide sequence of MAYV was determined in 2005 [78]. MAYV is part of the Semliki Forest Complex due to its antigenic composition and is phylogenetically close to other alphaviruses such as CHIKV, Sindbis, and Ross river viruses that induce an identical clinical illness in infected patients [4,15]. Its antigenic resemblance to CHIKV has been well described [72]. Nucleotide sequence homologies and recent phylogenetic analysis carried out on MAYV strains isolated from the Americas indicate the existence of three major phylogroups: disseminated (D), limited (L), and new (N) [79,80]. D is the more widely disseminated group and contains strains identified in several countries in the Americas (Trinidad and Tobago, Peru, French Guiana, Venezuela, and Bolivia), while the phylogroup limited (L) is found only in a few countries (Haiti and Brazil) [31]. A minor genotype, new (N), which is limited to only one known sequence, was isolated in 2010 in Peru [81].

## 7. Transmission Cycle

**Figure 5:**
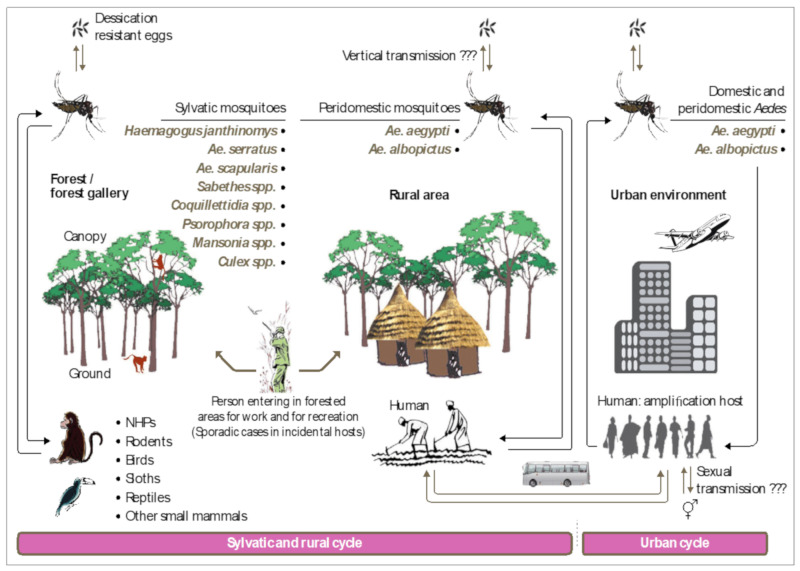
MAYV transmission cycles in endemic South and Central Americas. The list of arthropod and vertebrate species or groups mentioned in this figure may not be exhaustive due to the lack of data for certain regions. The figure was designed using Adobe Creative Cloud apps (https://www.adobe.com/creativecloud.html).

In MAYV-endemic regions in Latin America, a viral transmission cycle in the sylvatic environment is observed. This cycle mainly involves the mosquito *Haemagogus janthinomys* and non-human primates (NHP), considered to be the primary hosts of the virus [82,83,84] (Figure 5). To a lesser extent, this cycle may involve not only other vertebrates or secondary hosts (rodents, reptiles, etc.), whose role is not clearly identified, but also other types of mosquitoes such as *Mansonia*, *Culex*, *Sabethes*, *Aedes*, and *Psorophora* which may play an accessory or occasional role in this transmission cycle [10,26,85,86,87]. Due to both the zoophagous nature of *Haemagogus* spp. and their limited biotope in rural areas close to forests, humans are only sporadically infected [88]. At present, no animal reservoir of MAYV has been formally documented in Africa, Asia, and Europe. In urban areas, domestic mosquitoes are potential vectors of MAYV that could transmit the virus to humans under appropriate conditions. The introduction of MAYV into urban habitats and their establishment a domestic cycle is increasingly likely. If this process becomes a reality, this situation could be a real burden, not only for endemic regions but also on a global scale, especially when possible mutations of the virus will favor more efficient dissemination by domestic vectors [25]. In addition to this classical mode of transmission, there is also vertical transmission (passage of the virus from an infected female to her offspring), a mechanism by which the vector contains the virus for long periods of time, thereby constituting a true reservoir. Vertical transmission was revealed by the isolation of MAYV from two pools of adult *Ae*. aegypti which were collected from the egg stage in Brazil in 2017 [89]. Further studies are needed to identify the importance of vertical transmission during epidemics and the molecular mechanism that underlies viral maintenance in the vectors during unfavorable periods [90]. It is, however, clear that the effectiveness of this mechanism depends on climate change, geographical location, arbovirus genotype, and vector population dynamics [91]. Although this mechanism has been extensively reported for DENV, CHIKV, ZIKV, and Yellow fever virus infections [92,93,94,95], very few studies have addressed its role in MAYV and other arboviruses [89]. Airborne transmission of MAYV has also been reported among laboratory personnel [96].

## 8. MAYV Vectors: Field and Laboratory Observations

To incriminate a mosquito species as a vector of MAYV, two conditions must be met, preferably in this order: 1) first, the virus must be detected in natura from specimens of the species and 2) experiences in controlled conditions must provide evidence of its capacity to transmit the virus. Several entomological surveillances across the world have provided the suspicious involvement of many species but have not given enough formal evidence for the incrimination of a particular species. Indeed, any pathogen is likely to be detected in blood ingested by an arthropod [97]. On the other hand, although transmission experiments are able to clarify potential implications, the number of species to be tested for vector competence is limited by certain technical restrictions and this approach is not always relevant due to the lack of contact between the arthropod and humans in natura [98]. Therefore, the synthesis of all observations obtained from these studies will enable us to define the true MAYV vectors implicated in the viral transmission cycles (Figure 5).

### 8.1. Evidence from the Field

MAYV isolates have been obtained from diverse mosquito genera (*Haemagogus*, *Culex*, *Mansonia*, *Sabethes*, *Psorophora*, *Aedes*) (see Table 1). *Haemagogus* spp., especially *Hg*. *janthinomys*, appears to be the primary vector [84]. In 1957, a strain of MAYV was isolated from a pool (49 specimens) of the mosquito *Mansonia venezuelensis*, collected in northeastern Trinidad. This isolation was the first one recorded from arthropods collected in natura [99]. Subsequently, isolates were frequently obtained from *Hg*. *janthinomys* and less regularly from other mosquito species [83,100,101]. In addition, many MAYV isolates were reported in 1978 in Brazil from *Hg*. *janthinomys* [84]. *Cx*. *quinquefasciatus* mosquitoes found naturally infected with MAYV were also reported in Brazil [28]; nevertheless, their ability to transmit the virus is still unproven [102].

### 8.2. Vector Competence Studies

There are a few studies on vector competence for MAYV [26,98,103,104] and those carried out concern *Ae*. *aegypti* populations from Iquitos, Florida and French Polynesia, and *Ae*. *albopictus* from Brazil, Florida and Reunion [98,103,104,105,106]. These studies have shown that *Ae*. *albopictus* and *Ae*. *aegypti* are capable of transmitting MAYV. In addition, several other geographically divergent Anopheles species can transmit MAYV (Table 2) [98,103,104]. The latter observation suggests the possible involvement of some species of this genus in the amplification of MAYV in naïve areas. 

## 9. Major Urban MAYV Vectors: Extension and Risk to Developed and Developing Countries

### 9.1. Aedes aegypti

**Figure 6:**
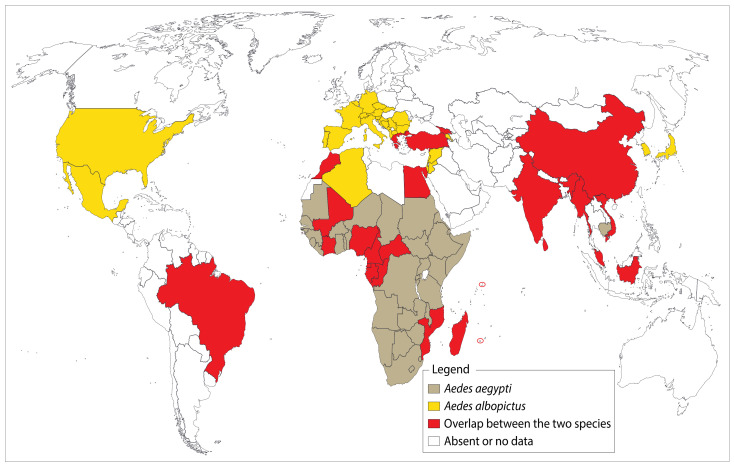
Worldwide distribution of *Ae*. *albopictus* and *Ae*. *aegypti*. The “tiger mosquito”, *Ae*. *albopictus*, has currently conquered several temperate regions including France, Italy, and Spain as shown on this map. The species is indeed a competent vector for several alphaviruses and flaviviruses which raises concerns when considering the occurrence of imported cases reported in the USA and Europe. On the other hand, *Ae*. aegypti is present in almost all tropical regions across the globe. On many occasions, the World Health Organization and the Rockefeller foundation have tried unsuccessfully to eradicate it. These two main urban vectors are not necessarily present throughout the countries/territories shaded in this map. ArcGIS Desktop V 10.5 software was used to generate this map (ESRI 2019, Redlands, CA, USA: Environmental Systems Research Institute; https://desktop.arcgis.com/en/).

The mosquito *Ae*. *aegypti* is a highly anthropophilic and extremely opportunistic species, with a nearly worldwide distribution. This is a domestic vector involved in arbovirus transmission at a global scale [1,107]. This vector has not yet been reported in European countries, except for some regions (Figure 6), although global warming could lead to their colonization in the near future. The delay of this phenomena will be determined particularly by global climate policies such as the Paris Agreement [108]. A few studies are have also dealt with climatic factors and mechanisms involved in the invasion and proliferation of this species [107,109,110,111].

### 9.2. Aedes albopictus

*Ae*. *albopictus*, commonly known as the “tiger mosquito”, is native to Southeast Asia. In recent years, the species has established itself in several regions across the world (Figure 6). In this process of invasion, scientists incriminate the transport of used tires which can harbor desiccation-resistant eggs. Following recent evidence from laboratory experiments on the ability of this species to transmit the virus, health officials expressed their concern and warned about the potential threat of a MAYV epidemic in areas already colonized by this species, as well as those threatened by its introduction.

## 10. Other Potential Vertebrate Hosts for MAYV

The presence of MAYV and/or anti-MAYV antibodies has been detected in vertebrate hosts in diverse localities in South America. In French Guiana, MAYV has been isolated from a wide variety of mammals, although their relevance in MAYV transmission is as yet unknown [112]. The virus has also been isolated from various other wild animals, including birds [113]. Anti-MAYV antibodies have been detected in marmosets (*Callithrix argentata*) as well.

## 11. MAYV Transmission in Nonhuman Primates

The rainforests in the Amazon region harbor diverse NHP species [114]. This diversity underlines the importance of identifying the level of involvement of each species in the bio-ecology of MAYV in order to acquire the necessary tools to better prepare or to avoid a worst-case scenario that could be generated by a possible amplification in human populations, starting from an enzootic cycle. As we are aware of these issues, it is important and urgent to carry out studies on the nature of NHPs present in this MAYV-endemic region. This lack of knowledge can be overcome by encouraging scientists who take an interest in this field and by setting up adequate infrastructure.

## 12. Emergence Mechanisms of Urban MAYV Transmission

Generally, the use of inappropriate vector control, coupled with the high density and the large distribution of urban *Aedes* spp., including *Ae*. *albopictus* and *Ae*. *aegypti*, is crucial in MAYV spreading [13]. Specifically, in low-income settings, it is mainly deforestation, unplanned policies concerning urbanization and infrastructure, as well as a lack of resources that benefit mosquito development and their contact with humans. Human movement may also increase the spread of MAYV, as shown for other similar vector-borne viruses [115,116,117,118]. Nevertheless, the incidence of disparities in transmission should be determined for vulnerable or minority communities most in need by improving access to high-quality telemedicine, increasing the use of mobile care and testing, in addition to reforming public health data practices, not only for MAYV, but also for other arboviruses as well.

## 13. Entomological Surveillance

At present, no surveillance system has been put in place for Mayaro fever and the first detections were obtained incidentally during investigations of febrile illnesses. During the epidemics of 1995 in Peru and 2008 in Brazil, results from surveillance efforts were based exclusively on reports provided by sentinel structures based on different techniques in use by the virology community. The occurrence of imported cases stresses the importance of global MAYV entomological surveillance. Such detection programs could provide an opportunity to bring together forces around the “one health” concept with an emphasis on vectors. The ambition, above all, would be to act more widely, more efficiently and more quickly on these issues in both the short and long term. Generally, entomological surveillance in a MAYV epidemic situation involves making an inventory of the mosquito species present at that time in the affected locality from the larval and adult stages, determining their abundance and, where appropriate, their distribution and potential involvement [15]. The latter can be provided by the results of attempts to isolate the virus from specimens collected in the field, including forests and human habitations. Until now, no efficient entomological surveillance of MAYV has been established. At present, the “human-landing catch” method is the preferred method for collecting those vectors likely to come into contact with humans, but unfortunately it is not appropriate in epidemic situations and is more expensive compared to other methods [119,120]. However, alternative methods (although they are less effective, but safer for the operator), through the use of different types of traps, can be used as well. In addition, surveillance of animal fauna (both wild and domestic) could provide additional details and thus allow us to refine the decision-making process to control MAYV. Taken together, the overall interpretation of the results from these surveys will provide a reliable estimation of the MAYV risk in the affected area.

## 14. Prevention and Control

In concrete terms, the prevention of Mayaro is contingent on the suppression of mosquito bites and, consequently, of human–vector contact. In the case of *Haemagogus* and *Aedes* vectors, whose peak activity coincides at the beginning or the end of the day, the use of insecticide-treated nets does not fully protect against these vectors. Because of the lack of a specific vaccine, the only effective way of managing the spread of the disease in endemic areas is vector control. This strategy, feasible in both domestic and peridomestic settings, focuses primarily on the suppression of mosquito breeding sites through the use of predators, larvicides, etc., which can be reinforced by global and multisectoral coordination across the fields of healthcare, community engagement and social communication [121]. The latter can be carried out among the local population and can take the form of awareness-raising actions about MAYV disease and individual protection measures against mosquito bites using different broadcasting channels and available media (TV, radio, social networks, etc.). Other vector control methods focusing on the elimination of adult stages by spraying with insecticides, particularly in epidemic situations, can also be implemented [84]. Unfortunately, the range of available molecules and formulations is limited, hence the importance of focusing on resistance levels in MAYV vector populations and understanding the underlying mechanisms. In the past, measures that were considered effective, based on the management of household water storage, were implemented in Brazil during the 1981 epidemic. Being among the less studied mosquito-borne diseases, MAYV is still neglected by health authorities and the general public across the world, specifically in non-endemic regions. Communities need to be aware of Mayaro fever epidemics, the damage the virus can cause to their health, and preventive measures, including cleaning their homes and cleaning water containers and the environment around their households. All necessary actions including social and behavior change should be taken in order to inspire community members to engage more strongly to prevent MAYV for a safer future. Surveillance, diagnosis of MAYV and vector control are the three main pillars in terms of prevention of the disease. In terms of vaccine research, efforts are being made and two promising vaccines are currently being developed and will hopefully soon be tested in clinical trials [122]. In order to respond to this crucial issue, the activities carried out in this field must be the culmination of a collective approach. At present, many questions about MAYV remain unresolved and require additional efforts by all multidisciplinary actors (virologists, entomologists, sociologists, statisticians, etc.) in order to find appropriate or alternative solutions.

## 15. Factors Associated with MAYV Emergence and Invasion

**Figure 7:**
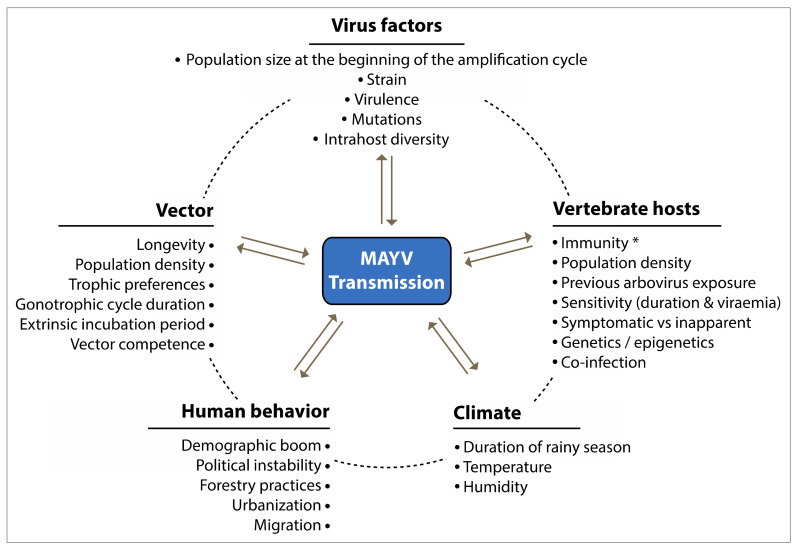
Schematic diagram showing the ecological factors that affect MAYV transmission. The dashed line and double arrows illustrate possible interactions between factors. * Population-level susceptibility. The figure was designed using Adobe Creative Cloud apps (https://www.adobe.com/creativecloud.html).

Nowadays, most outbreaks of MAYV are restricted to rural settings close to tropical forests, but a possible future urbanization of MAYV is to be expected, as suggested by the following observations [40,123]: (1) urbanization as a possible result of the virus being carried by travelers or infected birds [14,124]; (2) the strong homology between MAYV with CHIKV, an alphavirus that circulates in urban areas; (3) the frequent observation of MAYV cases near tropical cities in which *Ae. aegypti*, the principal urban vector, is endemic [15]; (4) results from laboratory studies showing that both *Ae. albopictus* [103,104,105] and *Ae. aegypti* [98,103,105] are able to transmit MAYV (Table 2). Features related to the emergence of MAYV include (1) anthropic disturbances in the ecosystem, notably caused by farming and agrobusiness activities, urbanization, a demographic boom, poor sanitary conditions, deforestation and human migration (Figure 7) [3,125,126,127], (2) the genetic mutation of the virus and its adaptation to other arthropod vectors [128], (3) the ability of these vectors to tolerate a lethal dose of major insecticides, as well as increased pathogen resistance to drugs [129] and, finally, (4) changing weather conditions that favor the invasion of mosquito vectors into new areas in which humans have not encountered the virus and have not yet acquired a protective immune response [2,130,131]. Ultimately, is it fair to state that changing climatic conditions are mainly responsible for the increased incidence and risk of viral spread to humans. These act in favor of the vectors that subsequently occupy hitherto unscathed areas and consequently allow the virus to emerge in these areas, as illustrated by the MAYV epidemic in Haiti in 2015. The positive correlation between the occurrence of epidemics and the peak density of vector populations during the wet season in tropical regions are further proof of the impact of climate on the transmission of MAYV [2,5,31,113].

## 16. Future Trends

Like CHIKV and ZIKV, MAYV could grow significantly and become one of the most widespread arboviruses on the planet. This scenario could be triggered by an evolution of the virus, changes in the host–vector relationship, etc. To anticipate possible adverse trends in Mayaro fever, there is a need to implement improved strategies and strengthen existing diagnostic platforms. The lack of optimal sampling methods for mosquitoes remains one of the major gaps in MAYV surveillance. The “human-landing collection” method remains the only effective method for the correct estimation of human–vector relationships, but its use is being questioned and debated because of ethical considerations [119,120]. For the coming years, it will therefore be urgent to think about implementation of alternative methods that can provide more perfect sampling data. Given the complexity of vector-borne diseases, experiments on vector competence for MAYV should include, in addition to species of the genera *Aedes* and *Haemagogus*, both *An*. *gambiae* and *Cx*. *quinquefasciatus*, which are species of great importance and whose presence is strongly dependent on human habitation. Studies on the efficacy of antiviral drugs and vaccines are generally supported by animal models, but not all models are suitable for a particular scientific question. In the case of MAYV, it is necessary to define which animal model (NHP, mouse, hamster, etc.) is suitable for vaccine research. For this purpose, we can learn from past years of alphavirus research and refer to available and relevant studies. Brazil, French Guyana, Peru, Colombia and Panama are among the regions in the world most affected by Mayaro disease. To help fight MAYV, it is necessary to combine simple community-based experiments with scientific innovation. Mobile phones have become important for disease mapping and surveillance through the use of short messaging services in affected areas. Adapting user-friendly innovations to the living conditions of people in remote locations can change the dynamics of disease control. The development of innovative scientific products from research and development takes time, but easy access to those innovations is needed to combat vector-borne diseases. Public–private partnerships, as well as university and national programs should be promoted to develop innovative tools for prevention. Technology is and will continue to be undoubtedly changing the way we monitor health information systems and discover new innovative diagnostic or control strategies against arboviruses. From sample collection, to diagnostics, to data sharing, technology has firmly taken its place in our day-to-day research activities.

## 17. Conclusions

Variabilities in susceptibility and ability to disseminate and transmit MAYV have been reported depending on the extrinsic incubation conditions, the virus strains used and the mosquito species tested. These variations in the expression of oral receptivity have been repeatedly described in natural populations of vector mosquitoes. Several factors, both intrinsic and extrinsic to the vectors, may explain these variations. Among these, possible interactions between the virus and the microbiota of the mosquito’s midgut seem to be particularly important, knowing that this symbiosis could provide important physiological functions to the host, including the synthesis of essential nutrients, resistance to infection and stimulation of the immune system. In many studies on the involvement of the immune system, bacteria have been used, but it was only very recently that their role in the immune response against virus infections was discovered. Differences in the expression of genes involved in the immune response are probably due to changes in the specific composition of the bacterial flora present in the midgut of the host mosquito and are important factors influencing the variation in transmission. To date, no data on potential vectors are available on this aspect. Therefore, for further analysis, studies should be carried out on the diversity and relative proportions of the different taxa of this midgut microbiota. Given that virus–host interactions are often accompanied by variations in viral proteins, resulting in the emergence of disease, it would be interesting to investigate the contribution of recombination phenomena to the natural evolution of MAYV strains and the epidemiological risk that recombinant strains may present. From a technical point of view, the identification and subsequent inactivation, by homologous recombination, of genes involved in the replication or adsorption of viral particles, for example, could significantly reduce transmission. It would also be helpful to improve our knowledge of the mechanisms underlying MAYV replication in mosquitos. In this respect, further studies providing valuable information on the basic ecology and spatiotemporal dynamics of vector abundance and their association with MAYV should be carried out. Transmission probability models could be used to estimate the natural periodicity of virus incidence cycles in each potential host. These predictive models will certainly make it possible to improve monitoring methods, thereby providing predictive capacity for the identification and early warning of emergence risks, as well as their control, by appropriate and accepted preventive methods. From the same perspective, two axes should be explored: (i) understanding how human contact with MAYV via vectors may increase with intensive use of forest resources; (ii) understanding how wildlife populations and communities, acting as reservoirs and potential vectors, respond to changes in the environment. These modifications will have an impact on wildlife populations, leading to the displacement of native forest species and the introduction of new ones capable of colonizing disturbed areas, resulting in the emergence of arboviruses, including MAYV, which were previously confined to the biotopes of natural reservoirs, thereby increasing the risk of contact with new mammalian and human hosts.

## Figures and Tables

**Table 1:** Mosquito species in which MAYV was detected in natura.

Country of Origin	Species	References
Brazil	*Aedes aegypti*	[28,89]
Panama	*Sabethes* spp.	[21]
Brazil	*Haemagogus janthinomys*	[20,83,84]
Colombia	*Aedes serratus*	[101]
Brazil	*Cx*. *quinquefasciatus*	[28]
Panama	*Cx*. *vomerifer*	[26]
Trinidad and Tobago	*Mansonia venezuelensis*	[99]
Colombia	*Psorophora ferox*	[101]
Colombia	*Psorophora albipes*	[101]

**Table 2:** Vector species found experimentally competent.

Country of Origin	Species	References
Iquitos, Peru	*Ae*. *aegypti*	[98,102,106]
Bora-Bora, French Polynesia	*Ae*. *aegypti*	[105]
Florida, USA	*Ae*. *aegypti*	[103]
Brazil	*Ae*. *albopictus*	[104]
La Reunion	*Ae*. *albopictus*	[105]
Florida, USA	*Ae*. *albopictus*	[103]
Trinidad and Tobago	*Ae*. *scapularis*	[86]
Virginia, USA	*An*. *freeborni*	[102]
Maryland, USA	*An*. *gambiae*	[102]
Virginia, USA	*An*. *quadrimaculatus*	[102]
Maryland, USA	*An*. *stephensi*	[102]

## References

[1] Semenza J.C., Suk J.E. (2018). Vector-borne diseases and climate change: A European perspective. FEMS Microbiol. Lett..

[2] Gould E.A., Higgs S. (2009). Impact of climate change and other factors on emerging arbovirus diseases. Trans. R. Soc. Trop. Med. Hyg..

[3] Weaver S.C., Reisen W.K. (2010). Present and future arboviral threats. Antiviral Res..

[4] Anderson C.R., Downs W.G., Wattley G.H., Ahin N.W., Reese A.A. (1957). Mayaro Virus: A New Human Disease Agent. Am. J. Trop. Med. Hyg..

[5] LeDuc J.W., Pinheiro F.P., da Rosa A.P.A.T. (1981). An Outbreak of Mayaro Virus Disease in Belterra, Brazil II. Epidemiology. Am. J. Trop. Med. Hyg..

[6] Schaeffer M., Gajdusek D.C., Lema A.B., Eichenwald H. (1959). Epidemic Jungle Fevers Among Okinawan Colonists in the Bolivian Rain Forest. Am. J. Trop. Med. Hyg..

[7] Carvalho C.A.M., Silva J.L., Oliveira A.C., Gomes A.M.O. (2017). On the entry of an emerging arbovirus into host cells: Mayaro virus takes the highway to the cytoplasm through fusion with early endosomes and caveolae-derived vesicles. PeerJ.

[8] Mezencio J.M.S., de Souza W., Fonseca M.E.F., Rebello M.A. (1990). Ultrastructural study of Mayaro virus replication in BHK-21 cells. Arch. Virol..

[9] Mezencio J.M.S., de Souza W., Fonseca M.E.F., Rebello M.A. (1989). Replication of Mayaro virus in Aedes albopictus cells: An electron microscopic study. Arch. Virol..

[10] Vasconcelos P.F., Travassos da Rosa A., Rodrigues S.G., Travassos da Rosa E.S., Dégallier N., Travassos da Rosa J.F. (2001). Inadequate management of natural ecosystem in the Brazilian Amazon region results in the emergence and reemergence of arboviruses. Cad. Saúde Pública.

[11] Pinheiro F.P., Freitas R.B., da Rosa J.F.T., Gabbay Y.B., Mello W.A., LeDuc J.W. (1981). An Outbreak of Mayaro Virus Disease in Belterra, Brazil I. Clinical and Virological Findings. Am. J. Trop. Med. Hyg..

[12] Izurieta R.O., DeLacure D.A., Izurieta A., Hoare I.A., Reina Ortiz M. (2018). Mayaro virus: The jungle flu. Virus Adapt. Treat..

[13] Kraemer M.U., Sinka M.E., Duda K.A., Mylne A.Q., Shearer F.M., Barker C.M., Moore C.G., Carvalho R.G., Coelho G.E., Van Bortel W. (2015). The global distribution of the arbovirus vectors Aedes aegypti and Ae. albopictus. eLife.

[14] Theilacker C., Held J., Allering L., Emmerich P., Schmidt-Chanasit J., Kern W.V., Panning M. (2013). Prolonged polyarthralgia in a German traveller with Mayaro virus infection without inflammatory correlates. BMC Infect. Dis..

[15] Tesh R.B., Watts D.M., Russell K.L., Damodaran C., Calampa C., Cabezas C., Ramirez G., Vasquez B., Hayes C.G., Rossi C.A. (1999). Mayaro Virus Disease: An Emerging Mosquito-Borne Zoonosis in Tropical South America. Clin. Infect. Dis..

[16] Phillips P.A., Lehmann D., Spooner V., Barker J., Tulloch S., Sungu M., Canil K.A., Pratt R.D., Lupiwa T., Alpers M.P. (1990). Viruses associated with acute lower respiratory tract infections in children from the Eastern Highlands of Papua New Guinea (1983–1985). Southeast Asian J. Trop. Med. Public Health.

[17] Acosta-Ampudia Y., Monsalve D.M., Rodríguez Y., Pacheco Y., Anaya J.-M., Ramírez-Santana C. (2018). Mayaro: An emerging viral threat?. Emerg. Microbes Infect..

[18] Aguilar-Luis M.A., del Valle-Mendoza J., Silva-Caso W., Gil-Ramirez T., Levy-Blitchtein S., Bazán-Mayra J., Zavaleta-Gavidia V., Cornejo-Pacherres D., Palomares-Reyes C., del Valle L.J. (2020). An emerging public health threat: Mayaro virus increases its distribution in Peru. Int. J. Infect. Dis..

[19] Pego P.N., Gomes L.P., Provance D.W., De Simone S.G. (2014). Mayaro Virus Disease. J. Hum. Virol. Retrovirol..

[20] Azevedo R.S.S., Silva E.V.P., Carvalho V.L., Rodrigues S.G., Neto J.P.N., Monteiro H.A.O., Peixoto V.S., Chiang J.O., Nunes M.R.T., Vasconcelos P.F.C. (2009). Mayaro Fever Virus, Brazilian Amazon. Emerg. Infect. Dis..

[21] Galindo P., Srihongse S., Rodaniche E.D., Grayson M.A. (1966). An Ecological Survey for Arboviruses in Almirante, Panama, 1959–1962. Am. J. Trop. Med. Hyg..

[22] Pinheiro F., Travassos da Rosa A., Freitas R., Travassos da Rosa J., Vasconcelos P. (1986). Aspectos clínico-epidemiológicos das arboviroses. Inst. Evandro Chagas.

[23] Seymour C., Peralta P.H., Montgomery G.G. (1983). Serologic Evidence of Natural Togavirus Infections in Panamanian Sloths and Other Vertebrates. Am. J. Trop. Med. Hyg..

[24] de Oliveira Mota M.T., Ribeiro M.R., Vedovello D., Nogueira M.L. (2015). Mayaro virus: A neglected arbovirus of the Americas. Future Virol..

[25] Mackay I.M., Arden K.E. (2016). Mayaro virus: A forest virus primed for a trip to the city?. Microbes Infect..

[26] Galindo P., Srihongse S. (1967). Transmission of Arboviruses to Hamsters by the Bite of Naturally Infected Culex (Melanoconion) Mosquitoes. Am. J. Trop. Med. Hyg..

[27] Muñoz M., Navarro J.C. (2012). Mayaro: A re-emerging Arbovirus in Venezuela and Latin America. Biomédica.

[28] Serra O.P., Cardoso B.F., Ribeiro A.L.M., dos Santos F.A.L., Slhessarenko R.D. (2016). Mayaro virus and dengue virus 1 and 4 natural infection in culicids from Cuiabá, state of Mato Grosso, Brazil. Mem. Inst. Oswaldo Cruz.

[29] Mourão M.P.G., de Souza Bastos M., de Figueiredo R.P., Gimaque J.B.L., dos Santos Galusso E., Kramer V.M., de Oliveira C.M.C., Naveca F.G., Figueiredo L.T.M. (2011). Mayaro Fever in the City of Manaus, Brazil, 2007–2008. Vector-Borne Zoonotic Dis..

[30] Halsey E.S., Siles C., Guevara C., Vilcarromero S., Jhonston E.J., Ramal C., Aguilar P.V., Ampuero J.S. (2013). Mayaro Virus Infection, Amazon Basin Region, Peru, 2010–2013. Emerg. Infect. Dis..

[31] Lednicky J., De Rochars V.M.B., Elbadry M., Loeb J., Telisma T., Chavannes S., Anilis G., Cella E., Ciccozzi M., Okech B. (2016). Mayaro Virus in Child with Acute Febrile Illness, Haiti, 2015. Emerg. Infect. Dis..

[32] Diaz L.A., Spinsanti L.I., Almiron W.R., Contigiani M.S. (2003). UNA virus: First report of human infection in Argentina. Rev. Inst. Med. Trop. São Paulo.

[33] Forshey B.M., Guevara C., Laguna-Torres V.A., Cespedes M., Vargas J., Gianella A., Vallejo E., Madrid C., Aguayo N., Gotuzzo E. (2010). Arboviral etiologies of acute febrile illnesses in Western South America, 2000–2007. PLoS Negl. Trop. Dis..

[34] Blohm G., Elbadry M.A., Mavian C., Stephenson C., Loeb J., White S., Telisma T., Chavannes S., Beau De Rochar V.M., Salemi M. (2019). Mayaro as a Caribbean traveler: Evidence for multiple introductions and transmission of the virus into Haiti. Int. J. Infect. Dis..

[35] Hassing R.-J., Leparc-Goffart I., Blank S.N., Thevarayan S., Tolou H., van Doornum G., van Genderen P.J. (2010). Imported Mayaro virus infection in the Netherlands. J. Infect..

[36] Gautret P., Cramer J.P., Field V., Caumes E., Jensenius M., Gkrania-Klotsas E., de Vries P.J., Grobusch M.P., Lopez-Velez R., Castelli F. (2012). Infectious diseases among travellers and migrants in Europe, EuroTravNet 2010. Eurosurveillance.

[37] Receveur M.C., Grandadam M., Pistone T., Malvy D. (2010). Infection with Mayaro virus in a French traveller returning from the Amazon region, Brazil, January, 2010. Eurosurveillance.

[38] Neumayr A., Gabriel M., Fritz J., Günther S., Hatz C., Schmidt-Chanasit J., Blum J. (2012). Mayaro Virus Infection in Traveler Returning from Amazon Basin, Northern Peru. Emerg. Infect. Dis..

[39] Llagonne-Barets M., Icard V., Leparc-Goffart I., Prat C., Perpoint T., André P., Ramière C. (2016). A case of Mayaro virus infection imported from French Guiana. J. Clin. Virol..

[40] Coimbra T.L.M., Santos C.L.S., Suzuki A., Petrella S.M.C., Bisordi I., Nagamori A.H., Marti A.T., Santos R.N., Fialho D.M., Lavigne S. (2007). Mayaro virus: Imported cases of human infection in São Paulo State, Brazil. Rev. Inst. Med. Trop. São Paulo.

[41] Talarmin A., Chandler L.J., Kazanji M., de Thoisy B., Debon P., Lelarge J., Labeau B., Bourreau E., Vié J.C., Shope R.E. (1998). Mayaro virus fever in French Guiana: Isolation, identification, and seroprevalence. Am. J. Trop. Med. Hyg..

[42] Arenívar C., Rodríguez Y., Rodríguez-Morales A.J., Anaya J.-M. (2019). Osteoarticular manifestations of Mayaro virus infection. Curr. Opin. Rheumatol..

[43] Dupuis-Maguiraga L., Noret M., Brun S., Grand R.L., Gras G., Roques P. (2012). Chikungunya Disease: Infection-Associated Markers from the Acute to the Chronic Phase of Arbovirus-Induced Arthralgia. PLoS Negl. Trop. Dis..

[44] Chow A., Her Z., Ong E.K.S., Chen J., Dimatatac F., Kwek D.J.C., Barkham T., Yang H., Rénia L., Leo Y.-S. (2011). Persistent Arthralgia Induced by Chikungunya Virus Infection is Associated with Interleukin-6 and Granulocyte Macrophage Colony-Stimulating Factor. J. Infect. Dis..

[45] Labadie K., Larcher T., Joubert C., Mannioui A., Delache B., Brochard P., Guigand L., Dubreil L., Lebon P., Verrier B. (2010). Chikungunya disease in nonhuman primates involves long-term viral persistence in macrophages. J. Clin. Invest..

[46] Noret M., Herrero L., Rulli N., Rolph M., Smith P.N., Li R.W., Roques P., Gras G., Mahalingam S. (2012). Interleukin 6, RANKL, and Osteoprotegerin Expression by Chikungunya Virus-Infected Human Osteoblasts. J. Infect. Dis..

[47] Morrison T.E., Oko L., Montgomery S.A., Whitmore A.C., Lotstein A.R., Gunn B.M., Elmore S.A., Heise M.T. (2011). A Mouse Model of Chikungunya Virus–Induced Musculoskeletal Inflammatory Disease: Evidence of Arthritis, Tenosynovitis, Myositis, and Persistence. Am. J. Pathol..

[48] Gardner J., Anraku I., Le T.T., Larcher T., Major L., Roques P., Schroder W.A., Higgs S., Suhrbier A. (2010). Chikungunya Virus Arthritis in Adult Wild-Type Mice. J. Virol..

[49] Tappe D., Pérez-Girón J.V., Just-Nübling G., Schuster G., Gómez-Medina S., Günther S., Muñoz-Fontela C., Schmidt-Chanasit J. (2016). Sustained Elevated Cytokine Levels during Recovery Phase of Mayaro Virus Infection. Emerg. Infect. Dis..

[50] Santiago F.W., Halsey E.S., Siles C., Vilcarromero S., Guevara C., Silvas J.A., Ramal C., Ampuero J.S., Aguilar P.V. (2015). Long-Term Arthralgia after Mayaro Virus Infection Correlates with Sustained Pro-inflammatory Cytokine Response. PLoS Negl. Trop. Dis..

[51] Santos F.M., Dias R.S., de Oliveira M.D., Costa I.C.T.A., de Souza Fernandes L., Pessoa C.R., da Matta S.L.P., Costa V.V., Souza D.G., da Silva C.C. (2019). Animal model of arthritis and myositis induced by the Mayaro virus. PLoS Negl. Trop. Dis..

[52] Churchman S.M., Ponchel F. (2008). Interleukin-7 in rheumatoid arthritis. Rheumatology.

[53] Yoo S.-A., Yoon H.-J., Kim H.-S., Chae C.-B., Falco S.D., Cho C.-S., Kim W.-U. (2009). Role of placenta growth factor and its receptor flt-1 in rheumatoid inflammation: A link between angiogenesis and inflammation. Arthritis Rheum..

[54] Camini F.C., da Silva Caetano C.C., Almeida L.T., da Costa Guerra J.F., de Mello Silva B., de Queiroz Silva S., de Magalhães J.C., de Brito Magalhães C.L. (2017). Oxidative stress in Mayaro virus infection. Virus Res..

[55] da Silva Caetano C.C., Camini F.C., Almeida L.T., Ferraz A.C., da Silva T.F., Lima R.L.S., de Freitas Carvalho M.M., de Freitas Castro T., Carneiro C.M., de Mello Silva B. (2019). Mayaro Virus Induction of Oxidative Stress is Associated With Liver Pathology in a Non-Lethal Mouse Model. Sci. Rep..

[56] Dhanwani R., Khan M., Alam S.I., Rao P.V.L., Parida M. (2011). Differential proteome analysis of Chikungunya virus-infected new-born mice tissues reveal implication of stress, inflammatory and apoptotic pathways in disease pathogenesis. Proteomics.

[57] Hosakote Y.M., Jantzi P.D., Esham D.L., Spratt H., Kurosky A., Casola A., Garofalo R.P. (2011). Viral-mediated Inhibition of Antioxidant Enzymes Contributes to the Pathogenesis of Severe Respiratory Syncytial Virus Bronchiolitis. Am. J. Respir. Crit. Care Med..

[58] Kayesh M.E.H., Ezzikouri S., Sanada T., Chi H., Hayashi Y., Rebbani K., Kitab B., Matsuu A., Miyoshi N., Hishima T. (2017). Oxidative Stress and Immune Responses During Hepatitis C Virus Infection in Tupaia belangeri. Sci. Rep..

[59] Gil L., Martínez G., Tápanes R., Castro O., González D., Bernardo L., Vázquez S., Kourí G., Guzmán M.G. (2004). Oxidative Stress in Adult Dengue Patients. Am. J. Trop. Med. Hyg..

[60] Cavalheiro M.G., Costa L.S.D., Campos H.S., Alves L.S., Assunção-Miranda I., Poian A.T.D., Cavalheiro M.G., Costa L.S.D., Campos H.S., Alves L.S. (2016). Macrophages as target cells for Mayaro virus infection: Involvement of reactive oxygen species in the inflammatory response during virus replication. An. Acad. Bras. Ciênc..

[61] de Castro-Jorge L.A., de Carvalho R.V.H., Klein T.M., Hiroki C.H., Lopes A.H., Guimarães R.M., Fumagalli M.J., Floriano V.G., Agostinho M.R., Slhessarenko R.D. (2019). The NLRP3 inflammasome is involved with the pathogenesis of Mayaro virus. PLOS Pathog..

[62] Yang Z., Min Z., Yu B. (2020). Reactive oxygen species and immune regulation. Int. Rev. Immunol..

[63] Chen W., Foo S.-S., Rulli N.E., Taylor A., Sheng K.-C., Herrero L.J., Herring B.L., Lidbury B.A., Li R.W., Walsh N.C. (2014). Arthritogenic alphaviral infection perturbs osteoblast function and triggers pathologic bone loss. Proc. Natl. Acad. Sci. USA.

[64] Rulli N.E., Rolph M.S., Srikiatkhachorn A., Anantapreecha S., Guglielmotti A., Mahalingam S. (2011). Protection from Arthritis and Myositis in a Mouse Model of Acute Chikungunya Virus Disease by Bindarit, an Inhibitor of Monocyte Chemotactic Protein-1 Synthesis. J. Infect. Dis..

[65] Bengue M., Ferraris P., Baronti C., Diagne C.T., Talignani L., Wichit S., Liegeois F., Bisbal C., Nougairède A., Missé D. (2019). Mayaro Virus Infects Human Chondrocytes and Induces the Expression of Arthritis-Related Genes Associated with Joint Degradation. Viruses.

[66] Torres J.R., Russell K.L., Vasquez C., Barrera R., Tesh R.B., Salas R., Watts D.M. (2004). Family Cluster of Mayaro Fever, Venezuela. Emerg. Infect. Dis..

[67] Figueiredo L.T.M., Nogueira R.M.R., Cavalcanti S.M.B., Schatzmayr H., da Rosa A.T., Figueiredo L.T.M., Nogueira R.M.R., Cavalcanti S.M.B., Schatzmayr H., da Rosa A.T. (1989). Study of two different enzyme immunoassays for the detection of Mayaro virus antibodies. Mem. Inst. Oswaldo Cruz.

[68] Earnest J.T., Basore K., Roy V., Bailey A.L., Wang D., Alter G., Fremont D.H., Diamond M.S. (2019). Neutralizing antibodies against Mayaro virus require Fc effector functions for protective activity. J. Exp. Med..

[69] da Rosa A.P.T., Vasconcelos P.F., da Rosa J.F.T. (1998). An Overview of Arbovirology in Brazil and Neighbouring Countries.

[70] Izurieta R.O., Macaluso M., Watts D.M., Tesh R.B., Guerra B., Cruz L.M., Galwankar S., Vermund S.H. (2011). Hunting in the Rainforest and Mayaro Virus Infection: An emerging Alphavirus in Ecuador. J. Glob. Infect. Dis..

[71] Strauss J.H., Strauss E.G. (1994). The alphaviruses: Gene expression, replication, and evolution. Microbiol. Rev..

[72] Karabatsos N. (1975). Antigenic Relationships of Group a Arboviruses by Plaque Reduction Neutralization Testing. Am. J. Trop. Med. Hyg..

[73] Fischer C., Bozza F., Merino X.J.M., Pedroso C., de Oliveira Filho E.F., Moreira-Soto A., Schwalb A., de Lamballerie X., Netto E.M., Bozza P.T. (2020). Robustness of Serologic Investigations for Chikungunya and Mayaro Viruses following Coemergence. mSphere.

[74] Calisher C.H., el-Kafrawi A.O., Mahmud M.I.A.-D., da Rosa A.P.T., Bartz C.R., Brummer-Korvenkontio M., Haksohusodo S., Suharyono W. (1986). Complex-specific immunoglobulin M antibody patterns in humans infected with alphaviruses. J. Clin. Microbiol..

[75] Lambert A.J., Martin D.A., Lanciotti R.S. (2003). Detection of North American Eastern and Western Equine Encephalitis Viruses by Nucleic Acid Amplification Assays. J. Clin. Microbiol..

[76] Firth A.E., Chung B.Y., Fleeton M.N., Atkins J.F. (2008). Discovery of frameshifting in Alphavirus 6K resolves a 20-year enigma. Virol. J..

[77] Zhang R., Kim A.S., Fox J.M., Nair S., Basore K., Klimstra W.B., Rimkunas R., Fong R.H., Lin H., Poddar S. (2018). Mxra8 is a receptor for multiple arthritogenic alphaviruses. Nature.

[78] Lavergne A., de Thoisy B., Lacoste V., Pascalis H., Pouliquen J.-F., Mercier V., Tolou H., Dussart P., Morvan J., Talarmin A. (2006). Mayaro virus: Complete nucleotide sequence and phylogenetic relationships with other alphaviruses. Virus Res..

[79] Powers A.M., Aguilar P.V., Chandler L.J., Brault A.C., Meakins T.A., Watts D., Russell K.L., Olson J., Vasconcelos P.F.C., Rosa A.T.D. (2006). Genetic Relationships among Mayaro and Una Viruses Suggest Distinct Patterns of Transmission. Am. J. Trop. Med. Hyg..

[80] Auguste A.J., Adams A.P., Arrigo N.C., Martinez R., da Rosa A.P.A.T., Adesiyun A.A., Chadee D.D., Tesh R.B., Carrington C.V.F., Weaver S.C. (2010). Isolation and Characterization of Sylvatic Mosquito-Borne Viruses in Trinidad: Enzootic Transmission and a New Potential Vector of Mucambo Virus. Am. J. Trop. Med. Hyg..

[81] Auguste A.J., Liria J., Forrester N.L., Giambalvo D., Moncada M., Long K.C., Morón D., de Manzione N., Tesh R.B., Halsey E.S. (2015). Evolutionary and Ecological Characterization of Mayaro Virus Strains Isolated during an Outbreak, Venezuela, 2010. Emerg. Infect. Dis..

[82] Mota M.T.O., Vedovello D., Estofolete C., Malossi C.D., Araújo J.P., Nogueira M.L. (2015). Complete Genome Sequence of Mayaro Virus Imported from the Amazon Basin to São Paulo State, Brazil. Genome Announc..

[83] Pinheiro F.P., LeDuc J.W. (1998). Mayaro virus disease. Arboviruses Epidemiol. Ecol..

[84] Hoch A.L., Peterson N.E., LeDuc J.W., Pinheiro F.P. (1981). An Outbreak of Mayaro Virus Disease in Belterra, Brazil III. Entomological and Ecological Studies. Am. J. Trop. Med. Hyg..

[85] Pauvolid-Corrêa A., Juliano R.S., Campos Z., Velez J., Nogueira R.M.R., Komar N., Pauvolid-Corrêa A., Juliano R.S., Campos Z., Velez J. (2015). Neutralising antibodies for Mayaro virus in Pantanal, Brazil. Mem. Inst. Oswaldo Cruz.

[86] Aitken T.H.G., Anderson C.R. (1959). Virus Transmission Studies with Trinidadian Mosquitoes. Am. J. Trop. Med. Hyg..

[87] Monath T.P. (1994). Dengue: The risk to developed and developing countries. Proc. Natl. Acad. Sci. USA.

[88] Abad-Franch F., Grimmer G.H., de Paula V.S., Figueiredo L.T.M., Braga W.S.M., Luz S.L.B. (2012). Mayaro Virus Infection in Amazonia: A Multimodel Inference Approach to Risk Factor Assessment. PLoS Negl. Trop. Dis..

[89] Maia L.M.S., Bezerra M.C.F., Costa M.C.S., Souza E.M., Oliveira M.E.B., Ribeiro A.L.M., Miyazaki R.D., Slhessarenko R.D. (2019). Natural vertical infection by dengue virus serotype 4, Zika virus and Mayaro virus in Aedes (Stegomyia) aegypti and Aedes (Stegomyia) albopictus. Med. Vet. Entomol..

[90] Gubler D.J. (2002). The Global Emergence/Resurgence of Arboviral Diseases as Public Health Problems. Arch. Med. Res..

[91] Bosio C.F., Thomas R.E., Grimstad P.R., Rai K.S. (1992). Variation in the Efficiency of Vertical Transmission of Dengue-1 Virus by Strains of Aedes albopictus (Diptera: Culicidae). J. Med. Entomol..

[92] Diallo D., Dia I., Diagne C.T., Gaye A., Diallo M., Higgs S., Vanlandingham D.L., Powers A.M. (2018). Chapter 4—Emergences of Chikungunya and Zika in Africa. Chikungunya and Zika Viruses.

[93] de Toni Aquino da Cruz L.C., Serra O.P., Leal-Santos F.A., Ribeiro A.L.M., Slhessarenko R.D., dos Santos M.A. (2015). Natural transovarial transmission of dengue virus 4 in Aedes aegypti from Cuiabá, State of Mato Grosso, Brazil. Rev. Soc. Bras. Med. Trop..

[94] Delatte H., Paupy C., Dehecq J.S., Thiria J., Failloux A.B., Fontenille D. (2008). Aedes albopictus, vector of chikungunya and dengue viruses in Reunion Island: Biology and control. Parasite Paris Fr..

[95] Mondet B., Vasconcelos P.F.C., Travassos da Rosa A.P.A., Travassos da Rosa E.S., Rodrigues S.G., Travassos da Rosa J.F.S., Bicout D.J. (2002). Isolation of Yellow Fever Virus from Nulliparous Haemagogus (Haemagogus) janthinomys in Eastern Amazonia. Vector-Borne Zoonotic Dis..

[96] Junt T., Heraud J.M., Lelarge J., Labeau B., Talarmin A. (1999). Determination of natural versus laboratory human infection with Mayaro virus by molecular analysis. Epidemiol. Infect..

[97] Gutiérrez-Bugallo G., Piedra L.A., Rodriguez M., Bisset J.A., Lourenço-de-Oliveira R., Weaver S.C., Vasilakis N., Vega-Rúa A. (2019). Vector-borne transmission and evolution of Zika virus. Nat. Ecol. Evol..

[98] Long K.C., Ziegler S.A., Thangamani S., Hausser N.L., Kochel T.J., Higgs S., Tesh R.B. (2011). Experimental Transmission of Mayaro Virus by Aedes aegypti. Am. J. Trop. Med. Hyg..

[99] Aitken T.H.G., Downs W.G., Anderson C.R., Spence L., Casals J. (1960). Mayaro Virus Isolated from a Trinidadian Mosquito, Mansonia venezuelensis. Science.

[100] Karabatsos N. (1985). International Catalogue of arthrOpod-Borne Viruses.

[101] Groot H., Morales A., Vidales H. (1961). Virus Isolations from Forest Mosquitoes in San Vicente de Chucuri, Colombia. Am. J. Trop. Med. Hyg..

[102] Brustolin M., Pujhari S., Henderson C.A., Rasgon J.L. (2018). Anopheles mosquitoes may drive invasion and transmission of Mayaro virus across geographically diverse regions. PLoS Negl. Trop. Dis..

[103] Wiggins K., Eastmond B., Alto B.W. (2018). Transmission potential of Mayaro virus in Florida Aedes aegypti and Aedes albopictus mosquitoes. Med. Vet. Entomol..

[104] Smith G.C., Francy D.B. (1991). Laboratory studies of a Brazilian strain of Aedes albopictus as a potential vector of Mayaro and Oropouche viruses. J. Am. Mosq. Control Assoc..

[105] Diop F., Alout H., Diagne C.T., Bengue M., Baronti C., Hamel R., Talignani L., Liegeois F., Pompon J., Vargas R.E.M. (2019). Differential Susceptibility and Innate Immune Response of Aedes aegypti and Aedes albopictus to the Haitian Strain of the Mayaro Virus. Viruses.

[106] Hotez P.J., Murray K.O. (2017). Dengue, West Nile virus, chikungunya, Zika—And now Mayaro?. PLoS Negl. Trop. Dis..

[107] Bhatt S., Gething P.W., Brady O.J., Messina J.P., Farlow A.W., Moyes C.L., Drake J.M., Brownstein J.S., Hoen A.G., Sankoh O. (2013). The global distribution and burden of dengue. Nature.

[108] Kraemer M.U., Reiner R.C., Brady O.J., Messina J.P., Gilbert M., Pigott D.M., Yi D., Johnson K., Earl L., Marczak L.B. (2019). Past and future spread of the arbovirus vectors Aedes aegypti and Aedes albopictus. Nat. Microbiol..

[109] Khormi H.M., Kumar L. (2014). Climate change and the potential global distribution of Aedes aegypti: Spatial modelling using geographical information system and CLIMEX. Geospatial Health.

[110] Caminade C., Medlock J.M., Ducheyne E., McIntyre K.M., Leach S., Baylis M., Morse A.P. (2012). Suitability of European climate for the Asian tiger mosquito Aedes albopictus: Recent trends and future scenarios. J. R. Soc. Interface.

[111] Medlock J.M., Avenell D., Barrass I., Leach S. (2006). Analysis of the potential for survival and seasonal activity of Aedes albopictus (Diptera: Culicidae) in the United Kingdom. J. Vector Ecol..

[112] de Thoisy B., Gardon J., Salas R.A., Morvan J., Kazanji M. (2003). Mayaro Virus in Wild Mammals, French Guiana. Emerg. Infect. Dis..

[113] Figueiredo L.T.M. (2007). Emergent arboviruses in Brazil. Rev. Soc. Bras. Med. Trop..

[114] Rylands A.B., Mittermeier R.A., Garber P.A., Estrada A., Bicca-Marques J.C., Heymann E.W., Strier K.B. (2009). The Diversity of the New World Primates (Platyrrhini): An Annotated Taxonomy. South American Primates: Comparative Perspectives in the Study of Behavior, Ecology, and Conservation.

[115] Ali S., Gugliemini O., Harber S., Harrison A., Houle L., Ivory J., Kersten S., Khan R., Kim J., LeBoa C. (2017). Environmental and Social Change Drive the Explosive Emergence of Zika Virus in the Americas. PLoS Negl. Trop. Dis..

[116] Bogoch I.I., Brady O.J., Kraemer M.U.G., German M., Creatore M.I., Brent S., Watts A.G., Hay S.I., Kulkarni M.A., Brownstein J.S. (2016). Potential for Zika virus introduction and transmission in resource-limited countries in Africa and the Asia-Pacific region: A modelling study. Lancet Infect. Dis..

[117] Stoddard S.T., Forshey B.M., Morrison A.C., Paz-Soldan V.A., Vazquez-Prokopec G.M., Astete H., Reiner R.C., Vilcarromero S., Elder J.P., Halsey E.S. (2013). House-to-house human movement drives dengue virus transmission. Proc. Natl. Acad. Sci. USA.

[118] Harrington L.C., Scott T.W., Lerdthusnee K., Coleman R.C., Costero A., Clark G.G., Jones J.J., Kitthawee S., Kittayapong P., Sithiprasasna R. (2005). Dispersal of the Dengue Vector Aedes aegypti within and between Rural Communities. Am. J. Trop. Med. Hyg..

[119] Higgs S., Vanlandingham D. (2015). Chikungunya Virus and Its Mosquito Vectors. Vector-Borne Zoonotic Dis..

[120] Achee N.L., Gould F., Perkins T.A., Reiner R.C., Morrison A.C., Ritchie S.A., Gubler D.J., Teyssou R., Scott T.W. (2015). A Critical Assessment of Vector Control for Dengue Prevention. PLoS Negl. Trop. Dis..

[121] Izurieta R.O., Macaluso M., Watts D.M., Tesh R.B., Guerra B., Cruz L.M., Galwankar S., Vermund S.H. (2009). Assessing Yellow Fever Risk in the Ecuadorian Amazon. J. Glob. Infect. Dis..

[122] Esposito D.L.A., da Fonseca B.A.L., Esposito D.L.A., da Fonseca B.A.L. (2017). Will Mayaro virus be responsible for the next outbreak of an arthropod-borne virus in Brazil?. Braz. J. Infect. Dis..

[123] de Figueiredo M.L.G., Figueiredo L.T.M. (2014). Emerging alphaviruses in the Americas: Chikungunya and Mayaro. Rev. Soc. Bras. Med. Trop..

[124] Calisher C.H., Gutierrez E., Maness K., Lord R.D. (1974). Isolation of Mayaro virus from a migrating bird captured in Louisiana in 1967. Bull. Pan Am. Health Organ. PAHO.

[125] Weaver S.C. (2013). Urbanization and geographic expansion of zoonotic arboviral diseases: Mechanisms and potential strategies for prevention. Trends Microbiol..

[126] Halstead S.B. (2019). Travelling arboviruses: A historical perspective. Travel Med. Infect. Dis..

[127] Cleton N., Koopmans M., Reimerink J., Godeke G.-J., Reusken C. (2012). Come fly with me: Review of clinically important arboviruses for global travelers. J. Clin. Virol..

[128] Mavian C., Rife B.D., Dollar J.J., Cella E., Ciccozzi M., Prosperi M.C.F., Lednicky J., Morris J.G., Capua I., Salemi M. (2017). Emergence of recombinant Mayaro virus strains from the Amazon basin. Sci. Rep..

[129] Beaty B.J., Black W., Eisen L., Flores A.E., García-Rejón J.E., Loroño-Pino M., Saavedra-Rodriguez K., Alison Mack R., Forum on Microbial Threats, Board on Global Health, Health and Medicine Division, National Academies of Sciences, Engineering, and Medicine (2016). The intensifying storm: Domestication of Aedes aegypti, urbanization of arboviruses, and emerging insecticide resistance. Global Health Impacts of Vector-Borne Diseases: Workshop Summary.

[130] Liu B., Gao X., Ma J., Jiao Z., Xiao J., Hayat M.A., Wang H. (2019). Modeling the present and future distribution of arbovirus vectors Aedes aegypti and Aedes albopictus under climate change scenarios in Mainland China. Sci. Total Environ..

[131] Kamal M., Kenawy M.A., Rady M.H., Khaled A.S., Samy A.M. (2018). Mapping the global potential distributions of two arboviral vectors Aedes aegypti and Ae. albopictus under changing climate. PLoS ONE.

